# Double Diabetes: A Converging Metabolic and Autoimmune Disorder Redefining the Classification and Management of Diabetes

**DOI:** 10.7759/cureus.80495

**Published:** 2025-03-12

**Authors:** Raj K Chaudhary, Obaid Ali, Amrendra Kumar, Abilesh Kumar, Anjum Pervez

**Affiliations:** 1 Department of Medicine, Jawaharlal Nehru Medical College, Bhagalpur, IND

**Keywords:** autoimmune dysfunction, beta-cell dysfunction, double diabetes, insulin resistance, metabolic syndrome, precision medicine

## Abstract

This review explores the pathophysiology, clinical implications, and management of double diabetes. The increasing prevalence of obesity, sedentary lifestyles, and genetic predisposition has blurred the difference between type 1 and type 2 diabetes, leading to diagnostic and therapeutic challenges. Double diabetes presents with overlapping symptoms from both diabetes types, making accurate diagnosis crucial. Biomarkers, such as C-peptide levels, autoantibody testing, and insulin resistance markers, help differentiate double diabetes from classic diabetes subtypes. Early intervention is necessary because of the condition's elevated risk of microvascular and macrovascular consequences, such as retinopathy, nephropathy, and cardiovascular disease. Effective management integrates pharmacological and lifestyle approaches. Metformin, sodium-glucose cotransporter 2 (SGLT2) inhibitors, glucagon-like peptide-1 (GLP-1) receptor agonists, and insulin therapy adjustments all boost glycemic control and metabolic results. Additionally, structured exercise, dietary modifications, and weight management are essential for reducing insulin resistance and preserving beta-cell activity. The potential of precision medicine, artificial intelligence (AI)-driven healthcare, and continuous glucose monitoring (CGM) offers promising advancements for personalized treatment strategies. Future research should focus on targeted immunotherapies, genetic profiling, and refined clinical guidelines to improve early detection and individualized treatment, with long-term outcomes. The review emphasizes the need for a multidisciplinary approach in managing double diabetes, ensuring early diagnosis, optimized treatment, and improved metabolic health to mitigate long-term complications.

## Introduction and background

Double diabetes occurs when type 1 and type 2 diabetes share characteristics that represent a complex metabolic health condition. The combination of both types leads to double diabetes when insulin-resistant type 1 patients show type 2 diabetes symptoms, or when type 2 patients demonstrate type 1 diabetes markers [1]. Traditional diabetes classifications become insufficient when treating double diabetes because it requires both insulin deficiency therapy and insulin resistance management. Scientific research demonstrates that double diabetes exists as a unique medical condition, because patients frequently exhibit combined metabolic and autoimmune processes within their bodies [2].

The current rise of obesity, together with inactive lifestyle patterns, complicates the medical classification of diabetes between type 1 and type 2 [3]. Healthcare professionals detected double diabetes when type 1 diabetic patients developed symptoms of type 2 diabetes, including obesity, hypertension, and dyslipidemia [4]. Health studies demonstrate that double diabetes is becoming increasingly prevalent among type 1 diabetic children who face weight gain in addition to metabolic syndrome [5]. The worldwide increase in childhood obesity indicates that diabetes progression may change as people grow older. Research indicates that insulin resistance happens in 25%-30% of type 1 diabetic patients, causing cardiovascular complications that typically affect type 2 diabetic patients [6].

The diagnostic category of double diabetes produces extensive clinical challenges since it creates specific barriers to treatment management. Medical staff needs to detect double diabetes because individual treatments for type 1 or type 2 diabetes might not work effectively in these patients. The improper diagnosis or late identification of double diabetes results in inadequate treatment outcomes that heighten the probability of developing nephropathy, together with retinopathy and cardiovascular disease [7]. Double diabetic patients face major challenges with controlling their blood glucose, which requires comprehensive healthcare treatment by several professionals, combining lifestyle changes with both insulin therapy and insulin sensitizers [8]. The worldwide increase in diabetes cases requires a deep comprehension of double diabetes to create better patient outcomes and develop optimal healthcare systems.

The review investigates double diabetes through an evaluation of the pathophysiology, epidemiological data, clinical expressions, and emerging treatment approaches. The review explores current research on double diabetes, focusing on its metabolic and autoimmune components, challenges in diagnosis, and the need for personalized treatment. It highlights the role of precision medicine, artificial intelligence (AI), and pharmacological advancements in improving patient outcomes. Addressing these complexities is crucial for improving early detection, individualized treatment strategies, and long-term metabolic health in double diabetes management.

## Review

Convergence of type 1 and type 2 diabetes leading to double diabetes

**Figure 1:**
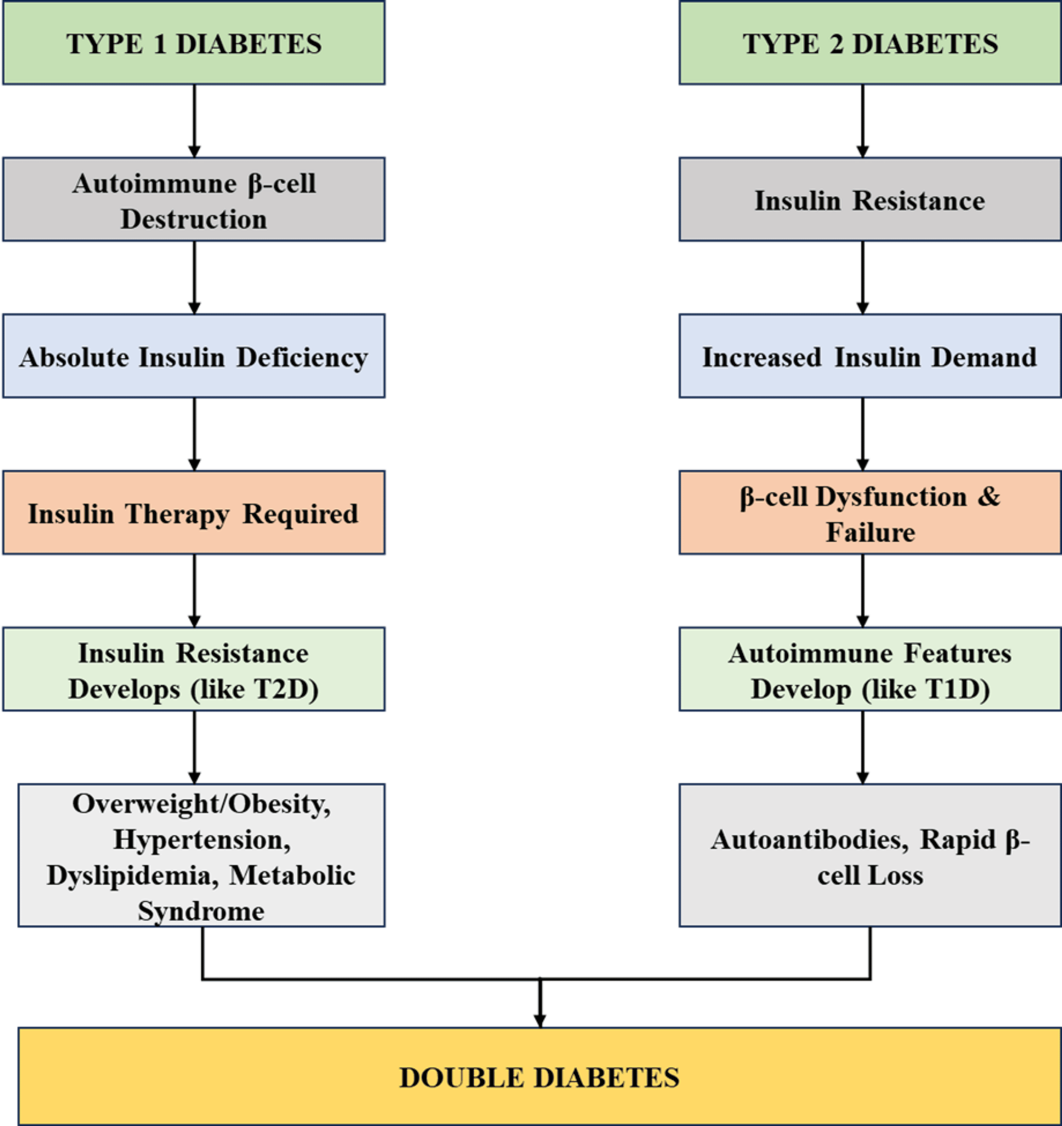
Convergence of Type 1 and Type 2 Diabetes Leading to Double Diabetes Image credit: The image has been created by the authors of this article

The combination of type 1 and type 2 diabetes characteristics produces double diabetes, which forms an intricate autoimmune and metabolic condition. The two fundamental processes through which double diabetes occurs are illustrated in Figure 1. Type 1 diabetic patients experience insulin resistance through a combination of obesity, sedentary habits, and genetic factors that delay their diabetes management, requiring extra medical support. As β-cell dysfunction worsens and autoimmune markers emerge, patients with type 2 diabetes become insulin-dependent. Such development resembles the insulin dependence of type 1 diabetes. Treatment and diagnosis, as well as management of this condition, become complex due to the convergence of these disease mechanisms. Early intervention, treatment planning, and improvement in metabolic outcomes for double diabetic patients require understanding these shared disease mechanisms.

Genetic predisposition and epigenetic influences

Double diabetes develops as a result of combined genetic elements and epigenetic regulatory mechanisms. Various genetic markers between type 1 and type 2 diabetes generate complex metabolic and autoimmune conditions that interact with insulin resistance. The chromosomal region 6p21 contains human leukocyte antigen (HLA)-DR/DQ, which enhances immune system regulation and raises susceptibility to beta-cell destruction caused by an autoimmune response. The HLA-DR/DQ gene expression shows changes through DNA methylation after environmental triggers, such as viral infections, activate the disease [8].

At chromosome 10q25, the transcription factor 7-like 2 (TCF7L2) type 2 diabetes risk gene controls beta-cell function as well as insulin secretion. Research shows TCF7L2 expression controls through histone adjustments and reacts to weight-related foods and obesity factors [9]. Another significant gene, INS (insulin gene) on chromosome 11p15, is crucial in insulin production. The INS gene expression receives control through epigenetic modifications, especially DNA methylation, that influence beta-cell function while affecting their response to metabolic stress [10].

The peroxisome proliferator-activated receptor gamma (PPARG) gene, located on the 3p25 chromosome in type 2 diabetes, controls lipid metabolism while simultaneously affecting insulin sensitivity. PPARG gene variants produce obesity-related insulin resistance because histone acetylation controls its functional activity [11]. DNA methylation and other epigenetic modifications influence the insulin secretion and potassium channel regulation functions of the type 2 diabetes gene potassium voltage-gated channel subfamily J member 11 (KCNJ11), which is located on chromosome 11p15.1 [12].

Type 1 diabetes risk and beta-cell destruction susceptibility depend on protein tyrosine phosphatase non-receptor type 22 (PTPN22), which serves as a primary immune regulatory gene found on chromosome 1p13. Studies suggest that the expression of PTPN22 becomes vulnerable to infections due to histone deacetylation effects. The combined genetic susceptibility and epigenetic control establish an individual's probability of developing double diabetes [13]. The key genetic markers, alongside their chromosomal positions, diabetes type, epigenetic modifications, and environmental triggers, are mentioned in Table 1.

**Table 1:** Genetic Predisposition and Epigenetic Influences in Double Diabetes HLA-DR/DQ: Human Leukocyte Antigen-DR/DQ; TCF7L2: Transcription Factor 7-Like 2; INS: Insulin Gene; PPARG: Peroxisome Proliferator-Activated Receptor Gamma; KCNJ11: Potassium Voltage-Gated Channel Subfamily J Member 11; PTPN22: Protein Tyrosine Phosphatase Non-Receptor Type 22

Gene/Marker	Chromosome Location	Associated Diabetes Type	Mechanism	Epigenetic Influence	Environmental Trigger	Impact on Beta Cells	Effect on Insulin Sensitivity
HLA-DR/DQ	6p21	Type 1	Autoimmune susceptibility	Methylation changes	Viral infections	Beta-cell destruction	Minimal
TCF7L2	10q25	Type 2	Beta-cell function	Histone modifications	Diet & Obesity	Reduced insulin secretion	High
INS	11p15	Type 1	Insulin gene regulation	DNA methylation	Prenatal nutrition	Impaired insulin production	Moderate
PPARG	3p25	Type 2	Lipid metabolism	Histone acetylation	High-fat diet	No direct impact	High
KCNJ11	11p15.1	Type 2	Potassium channel regulation	DNA methylation	Prenatal exposure to glucose	Impaired insulin release	Moderate
PTPN22	1p13	Type 1	Immune response modulation	Histone deacetylation	Infections	Autoimmune activation	Minimal

Role of insulin resistance in type 1 diabetic patients

Insulin resistance is a major metabolic issue that individuals with type 1 diabetes face, despite its historical association with type 2 diabetes. Individuals with type 1 diabetes may develop insulin resistance even with absolute insulin deficiency, worsening glucose control and cardiovascular risk [2]. Multiple factors produce insulin resistance, such as the combination of excessive body fat, genetic components, long-term high blood sugar, and swelling mechanisms within the body.

The prevalence of obesity stands as a primary reason that causes insulin resistance for patients who have type 1 diabetes. Results from studies show that people who have a higher BMI need larger insulin doses because their insulin sensitivity decreases, which leads to complicated treatment, along with elevated metabolic syndrome and cardiovascular risk [14,15]. Glucotoxicity, together with oxidative stress and inflammation, causes type 1 diabetic patients to develop more insulin resistance through the reduction of insulin signaling and glucose uptake [16,17]. Type 1 diabetic patients with elevated tumor necrosis factor-alpha (TNF-α) and interleukin-6 (IL-6) show worse insulin action due to their increased levels [18,19].

**Figure 2:**
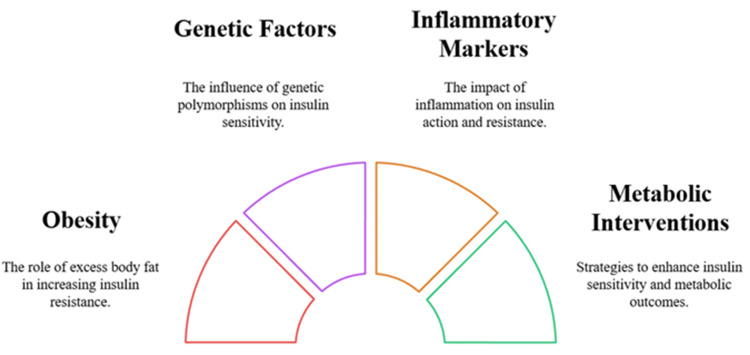
Key Factors Influencing Insulin Resistance in Type 1 Diabetes Management Image credit: The image has been created by the authors of this article

The insulin sensitivity levels of individuals with type 1 diabetes are influenced by the PPARG and TCF7L2 polymorphisms. Modifications of DNA structure, caused by dietary choices and life experiences, work to control metabolic responses [20,21]. The combination of metformin and sodium-glucose cotransporter 2 (SGLT2) inhibitors enhances both insulin sensitivity and metabolic results among patients who have double diabetes [22]. The early discovery of double diabetes, alongside specific intervention strategies, plays a vital role in preventing long-lasting medical complications and reaching optimal treatment results. Figure 2 shows the main parameters influencing insulin resistance in the management of type 1 diabetes.

Impact of autoimmune dysfunction on type 2 diabetes

Recent research findings demonstrate that autoimmune dysfunction is important for type 2 diabetes development, thus changing traditional disease classification methods. The diagnostic markers include insulin resistance, alongside beta-cell deterioration, but certain patients show evidence of autoimmune activity [23]. Pancreatic autoantibodies GADA (glutamic acid decarboxylase autoantibodies), IAA (insulin autoantibodies), and IA-2 (insulinoma-associated antigen-2) antibodies indicate that autoimmune destruction plays a role in the disease process [24].

Data shows that 10%-15% of type 2 diabetic patients carry pancreatic autoantibodies, which medical professionals diagnose as latent autoimmune diabetes in adults (LADAs) or double diabetes [25]. Type 1 diabetes differs from type 2 diabetes because autoimmunity in type 2 diabetes results in beta-cell decline, which increases insulin dependency progressively over time [26]. People with autoimmune markers experience quicker beta-cell deterioration, which leads them to need insulin therapy before antibody-negative patients [27].

The development of autoimmune dysfunction heavily depends on chronic inflammation. TNF-α, IL-6, and C-reactive protein (CRP) are pro-inflammatory cytokines that disrupt insulin signaling pathways while degrading beta-cell functioning abilities, disrupting normal blood glucose levels by permitting excessive hepatic glucose synthesis and impairing muscle glucose uptake [28,29]. The inflammatory process caused by autoimmunity leads to worsened insulin resistance and elevated cardiovascular dangers, which emerge as the main cause of death in these patients [30].

Genetic evaluations of HLA, PTPN22, and INS establish that the genes connect both autoimmune and metabolic diabetes types [31]. The disease progression of these conditions is influenced by environmental factors like diet and stress, together with infections, which modify epigenetic patterns [32].

The treatment of type 2 diabetes needs specific approaches when patients have autoimmune involvement. Patients with autoimmune markers show unresponsiveness to sulfonylureas and insulin secretagogues, which leads healthcare providers to consider glucagon-like peptide-1 (GLP-1) receptor agonists, dipeptidyl peptidase-4 (DPP-4) inhibitors, and insulin therapy as suitable alternatives [33].

Metabolic disturbances and beta-cell dysfunction

Metabolic disturbances give rise to both insulin resistance and impaired insulin secretion, together with beta-cell dysfunction, which advances diabetes progression. The combination of high blood sugar levels produces three detrimental effects on beta cells, which result in deteriorating blood glucose control. The prolonged elevation of blood glucose levels damages insulin signaling pathways, which causes insulin resistance to worsen [34].

Free fatty acids accumulating in the body through lipotoxicity reduce beta-cell function by generating problems with insulin granule release and raising both mitochondrial stress and inflammation levels, which drive insulin resistance. Reactive oxygen species produced by dysfunctional mitochondria cause beta-cell damage and DNA destruction, along with adenosine triphosphate (ATP) exhaustion, which speeds up metabolic decline. The unfolded protein response (UPR) is triggered by the endoplasmic reticulum's (ER) protein misfolding stress, which enhances both pro-inflammatory cytokine production and beta-cell death, thus accelerating diabetes development [35].

Chronic inflammation created by TNF-alpha, IL-6, and CRP interferes with insulin signaling and beta-cell functioning while raising cardiovascular danger levels. Type 2 diabetes advances faster when islet amyloid polypeptide (IAPP) builds up in the body because it damages beta cells and makes insulin resistance worse [36,37]. Initial treatment of glucotoxicity, lipotoxicity effects, and inflammatory processes, using blood sugar management solutions together with lifestyle changes, antioxidants, and anti-inflammatory drugs, helps delay disease progression and protect beta-cell functioning [38]. The influence of metabolic disturbances on beta-cell dysfunction appears in Table 2.

**Table 2:** Metabolic Disturbances and Their Impact on Beta-Cell Dysfunction ATP: Adenosine Triphosphate; ROS: Reactive Oxygen Species; TNF-α: Tumor Necrosis Factor-alpha; IL-6: Interleukin-6

Metabolic Disturbance	Primary Cause	Impact on Beta Cells	Effect on Insulin Secretion	Role in Insulin Resistance	Inflammatory Contribution	Associated Complications	Reversibility Potential
Glucotoxicity	Chronic hyperglycemia	Beta-cell apoptosis	Reduced insulin secretion	Increases insulin resistance	High (via oxidative stress)	Microvascular complications	Moderate
Lipotoxicity	Elevated free fatty acids	Beta-cell dysfunction	Impaired insulin granule release	Aggravates insulin resistance	Moderate (via cytokine release)	Non-alcoholic fatty liver disease	Low
Mitochondrial Dysfunction	Oxidative stress	Reduced ATP production	Impaired insulin exocytosis	Promotes insulin resistance	High (due to ROS production)	Neurodegeneration	Low
Endoplasmic Reticulum Stress	Protein misfolding	Beta-cell stress and apoptosis	Decreased insulin synthesis	Triggers inflammatory pathways	High (via unfolded protein response)	Diabetic nephropathy	Moderate
Chronic Inflammation	Cytokine overproduction	Beta-cell impairment	Impaired glucose-stimulated insulin secretion	Major contributor	Very high (TNF-α, IL-6 involvement)	Cardiovascular disease	Moderate
Amyloid Deposition	Accumulation of islet amyloid polypeptide	Beta-cell toxicity	Inhibits insulin release	Exacerbates insulin resistance	Moderate	Type 2 diabetes progression	Low

Epidemiological insights and risk factors in double diabetes

The increasing prevalence of double diabetes requires a thorough analysis of its risk factors, together with epidemiological data that involve insulin resistance alongside autoimmune dysfunction. The worldwide incidence of double diabetes continues to increase among youth because lifestyle changes and environmental elements affect their health. Scientific studies demonstrate that type 1 diabetic patients possess insulin resistance features in about 30% of cases, combined with obesity and metabolic syndrome, and type 2 diabetic patients show pancreatic autoantibodies that support an autoimmune origin [39]. The traditional separation between type 1 and type 2 diabetes recently became less evident because younger people face growing risks of obesity and inactivity, thus creating a new diabetes spectrum [40].

The pathophysiology and epidemiology of double diabetes strongly depend on lifestyle and environmental elements. Type 1 diabetic patients who are obese develop worse glycemic control and need more insulin because obesity acts as a major risk factor for insulin resistance [41]. Poor food choices, particularly processed carbohydrates, together with saturated fats and sugary beverages, create metabolic problems while causing insulin resistance in the body [42]. Early-life environmental exposures to maternal obesity and gestational diabetes put people at higher risk of developing beta-cell dysfunction and insulin resistance, which leads to a later-life double diabetes diagnosis [43].

The epidemiology of double diabetes is influenced by both genetic and ethnic variations of susceptibility. People who belong to the ethnic groups of Hispanic and African American people, along with those of South Asian and indigenous descent, exhibit natural tendencies toward insulin resistance and type 2 diabetes, which raises their chances of developing double diabetes when combined with autoimmune conditions [44]. The heterogeneity of double diabetes results from genetic variants that affect insulin resistance and autoimmune responses through HLA, TCF7L2, PTPN22, and INS genes [45]. The epigenetic modifications triggered by environmental elements, including diet, physical activity, and stress patterns, serve to control metabolic and immune functions, which affect the risk of double diabetes [46].

Clinical features and diagnosis

The diagnosis of double diabetes needs careful assessment because patients present symptoms that connect both type 1 and type 2 diabetes. The standard diagnosis indicators for type 2 diabetes, combined with type 1 diabetes features, include polyuria, polydipsia, and polyphagia (three Ps), alongside possible weight loss from type 1 diabetes [47,48].

The multiple ways through which double diabetes presents create difficulties in diagnosing the condition. The combination of insulin resistance in obese patients with type 1 diabetes can lead to a misdiagnosis of type 2 diabetes, yet type 2 diabetic patients with autoimmune markers require early insulin therapy because their beta-cell function declines rapidly [49]. The diagnostic challenges are summarized in Table 3, which presents clinical features and biomarkers, as well as laboratory criteria.

**Table 3:** Clinical Features and Diagnostic Markers of Double Diabetes DKA: Diabetic Ketoacidosis; HOMA-IR: Homeostatic Model Assessment of Insulin Resistance; IA-2: Islet Antigen-2 Autoantibodies; ZnT8: Zinc Transporter 8 Autoantibodies; GADA: Glutamic Acid Decarboxylase Autoantibodies; LADA: Latent Autoimmune Diabetes in Adults; HbA1c: Hemoglobin A1c (Glycated Hemoglobin); LDL: Low-Density Lipoprotein (Bad Cholesterol); TG: Triglycerides; HDL: High-Density Lipoprotein (Good Cholesterol); CGM: Continuous Glucose Monitoring

Clinical Feature	Overlapping Symptoms	Diagnostic Challenge	Diagnostic Criteria	Biomarkers	Laboratory Findings
Polyuria & Polydipsia	In both	It can be misattributed to either type	Frequent thirst & urination	C-peptide levels	Low in Type 1, Normal/High in Type 2
Unintended Weight Loss	More common in Type 1 but can occur in Type 2	Obesity in Type 2 may mask weight loss	Unexplained weight loss	Insulin autoantibodies	Positive in autoimmune involvement
Hyperglycemia	In both	Severity varies with insulin resistance	Fasting glucose >126 mg/dL or HbA1c >6.5%	HbA1c	Elevated in both
Ketoacidosis	More common in Type 1 but can occur in insulin-deficient Type 2	DKA risk in misdiagnosed Type 2 patients	Elevated ketones & acidosis	Beta-hydroxybutyrate	High in ketoacidosis
Insulin Resistance	Present in Type 2, emerging in Type 1 with obesity	Difficult to detect in early stages	HOMA-IR score & fasting insulin	Fasting insulin levels	High in Type 2, Normal/Low in Type 1
Dyslipidemia	Common in Type 2, emerging in Type 1 with metabolic syndrome	Often underdiagnosed in Type 1	Elevated LDL & triglycerides	Lipid profile	High LDL & TG, Low HDL
Autoimmune Markers	Absent in classic Type 2, but present in LADA/Double Diabetes	Overlap with late-onset Type 1	GADA, IA-2, ZnT8 testing	Autoantibodies	Positive in autoimmune diabetes
Beta-Cell Function Decline	Occurs in both, more rapid in Type 1	Difficult to differentiate in early stages	C-peptide testing	C-peptide levels	Low in Type 1, Declining in LADA
Glycemic Variability	Fluctuations in glucose levels	More in Type 1 but can occur in Type 2	Continuous glucose monitoring (CGM)	Time-in-range analysis	Highly variable in Type 1, More stable in Type 2

A correct diagnosis depends on C-peptide testing because it establishes the functional state of beta cells. The detection of type 1 diabetes appears through C-peptide test results below the threshold, yet insulin resistance typically shows normal-to-high levels, although double diabetes features declining C-peptide levels [50]. The assessment process benefits from additional markers of insulin resistance, which include HOMA-IR, fasting insulin, low-density lipoprotein (LDL) cholesterol, triglycerides, and inflammatory cytokines (TNF-α and IL-6) [51,52]. The continuous glucose monitoring (CGM) system helps patients reveal information about glycemic variability, since this data aids doctors in identifying insulin-deficient patients [52].

Health risks and associated complications in double diabetes

The combination of diabetes mellitus types 1 and 2 creates serious cardiovascular dangers because metabolic syndrome and cardiovascular diseases cause greater death rates, along with complications. Those who suffer from insulin resistance, together with autoimmunity, develop higher probabilities of coronary artery disease, stroke, heart failure, atherosclerosis, hypertension, and dyslipidemia [53]. Urgent medical intervention becomes necessary because chronic low-grade inflammation, together with endothelial dysfunction, speeds up cardiovascular breakdown. Metabolic syndrome components, such as central obesity and hypertension, together with dyslipidemia and insulin resistance, degrade both blood glucose management and heart health [54].

Patients with double diabetes also experience higher rates of microvascular and macrovascular complications. Progressive small blood vessel damage occurs from diabetic retinopathy, nephropathy, and neuropathy because of chronic hyperglycemia, together with oxidative stress and inflammatory responses [55]. The fast deterioration of renal function in diabetic nephropathy happens through two mechanisms, including beta-cell autoimmune damage and insulin-resistant hyperfiltration [56]. Additionally, peripheral artery disease and stroke severity increase due to arterial stiffening, platelet dysfunction, and pro-inflammatory cytokine activity [57]. Medical teams need to implement aggressive cardiovascular risk management because these complications lead to a significant reduction in patient life expectancy.

Mental health and the overall quality of life represent significant problems for these individuals. Patients who have double diabetes face more severe complications of diabetes distress, anxiety, and depression because they must manage insulin resistance, together with autoimmune dysfunction [58]. Those patients who experience burnout and treatment fatigue, together with complications-related anxiety, demonstrate lower rates of medical advice and lifestyle intervention compliance [59]. The extensive nature of diabetes care, with its ongoing observations and long-term medical complications, leads to emotional distress, which diminishes patients' quality of life [60]. The successful treatment of patients depends on psychological assistance, along with structured counseling and self-care programs, to achieve better patient results and improved well-being.

Pharmacological approaches in double diabetes management

Patients with double diabetes need insulin therapy combined with additional medications to treat the deficiency and resistance of insulin in their bodies. The main insulin therapy for significant beta-cell dysfunction is basal-bolus insulin therapy, yet each patient requires separate dose adjustments to manage hypoglycemic episodes and weight gain risks [61].

The administration of metformin produces beneficial effects for patients with type 1 diabetes features and insulin resistance because it boosts glucose uptake, along with decreased hepatic glucose production, which helps lower insulin needs and enhances metabolic measurements [62]. The medication thiazolidinediones (e.g., pioglitazone) boosts peripheral insulin sensitivity, but doctors need to exercise caution because these drugs can cause fluid retention, together with cardiovascular complications [63].

**Figure 3:**
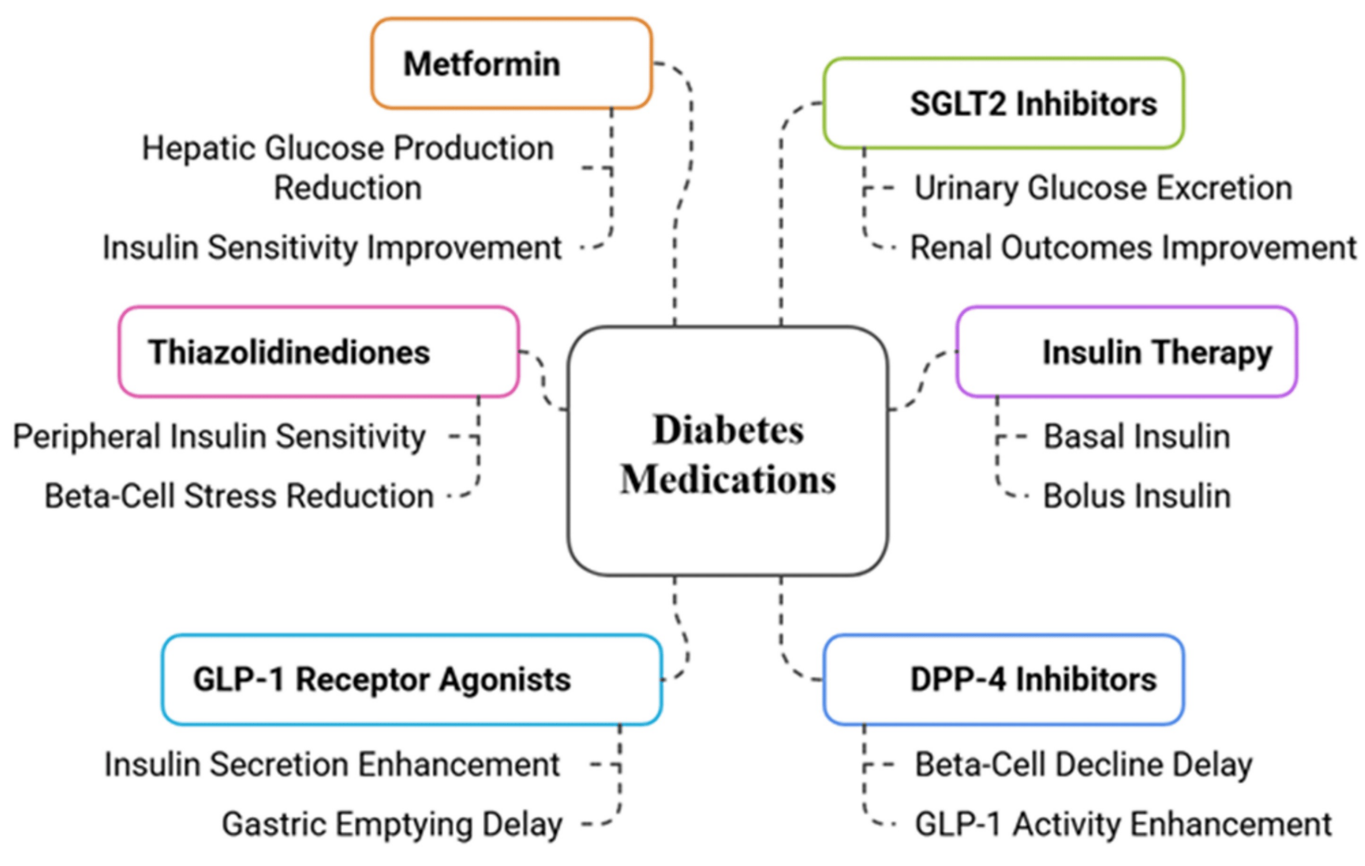
Pharmacological Approaches in Double Diabetes Management Image credit: The image has been created by the authors of this article SGLT2: Sodium-Glucose Cotransporter 2; GLP-1: Glucagon-Like Peptide-1; DPP-4: Dipeptidyl Peptidase-4

Liraglutide and semaglutide, among GLP-1 receptor agonists, help release insulin and restrain glucagon, while assisting in weight reduction and delivering heart protection for insulin-resistant and overweight patients [64]. Empagliflozin and dapagliflozin, among SGLT2 inhibitors, decrease blood glucose through kidney-based glucose elimination and simultaneously protect heart and kidney health. Euglycemic ketoacidosis poses a risk for patients who lack insulin, so they require special attention [65]. The DPP-4 inhibitors sitagliptin and linagliptin improve GLP-1 function to control blood glucose levels without causing hypoglycemia events and maintain beta-cell functionality [66]. Double diabetes treatment needs customized methods that combine insulin therapy with pharmaceutical medications chosen based on metabolic characteristics, heart health risks, and pancreatic cell functions. The pharmacological approaches with their clinical implications are presented in Figure 3.

Lifestyle modifications for improved metabolic health

Lifestyle modifications function as the essential approach for double diabetes management by treating both insulin resistance and beta-cell dysfunction. Strategic physical exercise, along with nutritional adjustments, leads to powerful enhancements of diabetic blood glucose management, together with better insulin response and better heart health. The medical treatment of diabetes should combine nutritional foods with monitored carbohydrate intake to achieve metabolic equilibrium. Studies prove that the Mediterranean and low-GI dietary patterns improve insulin sensitivity, decrease inflammation, and lower cardiovascular risk [67].

Nutritious foods containing fiber, combined with lean proteins and healthy fats, stop rapid glucose changes while stabilizing metabolism. The removal of processed carbohydrates, together with trans fats and unnecessary saturated fats, supports decreased insulin resistance and prevents weight gain [68]. Patients with double diabetes and hypertension should follow the DASH (Dietary Approaches to Stop Hypertension) dietary plan because it leads to a better lipid profile, along with blood pressure enhancement [69]. Engaging in routine physical exercise leads to better insulin response and a reduction in abdominal body fat, while improving heart health. Glucose metabolism improves, while insulin requirements decrease, when patients perform aerobic exercises, such as walking, cycling, and swimming, alongside resistance training methods, like weightlifting or bodyweight activities [70].

Two sessions of resistance training and 150 minutes of moderate aerobic activity per week are the bare minimum needed for metabolic health [71]. Weight management stands as an essential requirement for people who have obesity, together with insulin resistance. Weight reduction between 5% and 10% leads to substantial improvements in glucose control, together with better cardiovascular health. The combination of controlled intermittent fasting and calorie restriction has been proven to decrease insulin resistance and beta-cell stress levels, according to research [72]. Cost-control management plans that unite lifestyle interventions, CGM-feedback systems, and digital health platforms lead patients to better adhere and succeed over the long term with dual-diabetes management.

Personalized medicine approach in double diabetes

Through personalized medicine approaches, the management of double diabetes has evolved through individual-specific genetic profiles and metabolic characteristics, as well as phenotypic features. Early case identification happens through precision medicine and genetic profiling, while personalized treatment plans are created through understanding genetic aspects of insulin resistance and autoimmune dysfunction [73]. The development of double diabetes depends heavily on genetic markers, which include HLA variants, together with TCF7L2 polymorphisms and PTPN22 mutations. Genetic testing allows medical professionals to sort patients so they can start insulin treatment immediately for people with beta-cell deterioration and enhance insulin-sensitizing medications for those with insulin resistance [74].

The rising presence of AI and machine learning (ML) applications helps doctors with diabetes management by predicting glycemic variations, treatment optimization, and clinical decisions. AI algorithms process insulin pump data and lifestyle patterns, with CGM information, to provide dynamic adjustments for adjusting insulin doses and dietary suggestions that occur in real time [71]. ML enables the detection of early complications through its detection of diabetic nephropathy, together with diabetic retinopathy, thus facilitating preventive measures. Through risk stratification tools powered by AI, it becomes possible to establish which high-risk patients will gain the most benefit from GLP-1 receptor agonists or SGLT2 inhibitors, through the assessment of cardiovascular and metabolic data points [73].

The use of personalized medicine in double diabetes includes metabolite detection with AI and microbiome assessment, in addition to treatment algorithms that focus on individual lifestyle needs to build total patient care approaches. The advancement of precision medicine through genomics, digital health technologies, and AI analytics systems will boost early detection and optimized treatment and reduce complications in managing double diabetic patients.

## Conclusions

This review analyzed double diabetes pathophysiology, epidemiological data, clinical outcomes, and treatment strategies. The condition is becoming more prevalent due to obesity, sedentary lifestyles, and genetic factors. Early detection and targeted treatment approaches are essential. Double diabetes presents diagnostic challenges. Biomarker analysis, C-peptide levels, and autoantibody testing help differentiate it from standard diabetes types. Patients face a higher risk of both microvascular and macrovascular complications. Effective treatment requires a combination of insulin therapy, metformin, GLP-1 receptor agonists, or SGLT2 inhibitors. Lifestyle modifications, including structured exercise and dietary adjustments, are also necessary. Future advancements in AI-driven healthcare and CGM may enhance treatment options. Research should focus on refining diagnostic methods, exploring immunotherapies, and utilizing genetic testing for early detection. Sustained metabolic health in double diabetic patients remains a key priority.
